# Clinical, Demographic, and Angiographic Profiles of Amarnath Pilgrims Presenting With Acute Coronary Syndrome

**DOI:** 10.7759/cureus.58820

**Published:** 2024-04-23

**Authors:** Aamir Rashid, Sameer Purra, Shahood A Kakroo, Imran Hafeez, Ajaz A Lone, Hilal Rather

**Affiliations:** 1 Department of Cardiology, Sher-i-Kashmir Institute of Medical Sciences, Srinagar, IND

**Keywords:** amarnath pilgrims, mortality, myocardial infarction, acute coronary syndrome, high altitude

## Abstract

Introduction: The challenges posed by high altitude are particularly significant in terms of cardiovascular health. There are currently no data available on acute coronary syndrome (ACS) among Amarnath pilgrims. The objective of this study was to investigate the clinical and angiographic profiles of ACS among Amarnath pilgrims, focusing on demographic characteristics, risk factors, types of ACS, clinical presentation, angiographic findings, and in-hospital outcomes. By examining these aspects, we aimed to provide insights into the unique challenges faced by pilgrims during their spiritual journey and to identify potential strategies for improving the prevention and management of ACS in this population.

Methods: This was a hospital-based, prospective, observational study that included patients who had participated in the pilgrimage and presented with ACS between 2022 and 2023.

Results: Sixty patients were recruited for the study, with a mean age of 51.19 ± 11.17 years. Of these, 43 (71.7%) were male. Risk factors identified in the study included hypertension in 35 (58.3%), smoking in 23 (38.3%), diabetes mellitus in 18 (30%), and dyslipidemia in 25 (41.6%) patients. ST-elevation myocardial infarction (STEMI) was present in 46 (76.66%) patients, Anterior wall myocardial infarction (AWMI) occurred in 29 (48.3%), inferior wall myocardial infarction (IWMI) in 15 (25%), and high lateral wall myocardial infarction (HLWMI) in two (3.3%) patients. Of the 60 patients, 19 (31.6%) were in Killip class I, 16 (26.6%) were in class II, and 25 (41.6%) were in classes III or IV. The average time from the onset of symptoms to hospitalization was 7.6 ± 3.1 hours, significantly higher in those with Killip class III or IV (9.3 ± 3.6 vs. 5.4 ± 2.7 hours, p = 0.01). There were nine (15%) in-hospital deaths, and in the multivariate analysis, advanced Killip class (p = 0.04) and delays in hospitalization of more than six hours (p = 0.03) were found to be significant predictors of mortality.

Conclusion: In conclusion, 40% of patients presented in the advanced Killip class, and 15% experienced in-hospital mortality. The average time from the onset of symptoms to hospitalization was significantly higher for those categorized in the advanced Killip classes. Our study highlights a significant association between advanced Killip class, delay in hospitalization, and in-hospital mortality among Amarnath pilgrims with ACS, underscoring the importance of timely intervention. It is recommended that appropriate measures be taken to improve patient outcomes in these cases.

## Introduction

High-altitude locations have a terrestrial elevation at which oxygen hemoglobin saturation drops below 90%, which is typically around 2,500 meters [1]. Above this altitude, the body undergoes a series of pulmonary and cardiovascular adaptations to maintain adequate oxygenation. These adaptations involve increased pulmonary ventilation and cardiac output, primarily driven by a higher heart rate [2-5]. The cave of Baba Amarnath Ji, standing at 130 ft and 13,000 ft (3,882 meters) in the north Indian state of Jammu and Kashmir, is visited by thousands of devotees each year for a six-week period starting in June, in a pilgrimage known as the Amarnath pilgrimage.

The cave is situated at an elevation of 3,888 meters in a narrow gorge at the far end of the Lidder Valley in south Kashmir. There are two routes available to pilgrims: the longer route through Pahalgam (414 km from Jammu) and the relatively shorter Baltal route (363 km from Jammu). The combination of overcrowding, physical exertion, and high altitude poses significant health risks to the pilgrims. Recently, cardiovascular disease has emerged as one of the primary health concerns for these individuals. Our center is the leading cardiac center in the Union Territory and serves as the primary referral hospital for patients with acute coronary syndrome (ACS). Each year, we have observed that a substantial number of Amarnath pilgrims present to our hospital with ACS, displaying more severe symptoms and worse outcomes compared to other ACS patients. This observation led to the initiation of our study.

Despite research on high-altitude medicine that has investigated these adaptations [2-5], there are still gaps in comprehending ACS among Amarnath pilgrims. Previous research on ACS among Hajj pilgrims has revealed a correlation with an increased incidence of complications and adverse results. The suboptimal in-hospital outcomes among pilgrim patients may be attributable to the severe physical and emotional strain, as well as the delays in seeking medical assistance and undergoing timely revascularization. While there is some data on acute mountain sickness, including high-altitude pulmonary and cerebral edema, among Amarnath pilgrims [6], there is no information available on the clinical and demographic profiles of those presenting with ACS. The objective of this study was to investigate the clinical and angiographic profile of ACS among Amarnath pilgrims, focusing on demographic characteristics, risk factors, types of ACS, clinical presentation, angiographic findings, and in-hospital outcomes. By examining these aspects, we aimed to provide insights into the unique challenges faced by pilgrims during their spiritual journey and to identify potential strategies for improving the prevention and management of ACS in this population.

## Materials and methods

Study design

This was a hospital-based, prospective, observational study involving 60 patients enrolled in the 2022 and 2023 Amarnath pilgrimages. The choice of an observational study design for investigating ACS among Amarnath pilgrims was primarily driven by practical considerations and ethical constraints, such as the logistical challenges and ethical concerns associated with conducting a randomized controlled trial or interventional study during the dynamic and transient nature of the pilgrimage season. Besides, an observational study design allows for the collection of real-world data on pilgrims presenting with ACS during their journey to the Amarnath cave, facilitating the examination of natural patterns, risk factors, clinical outcomes, and healthcare utilization without interfering with the pilgrims' experiences.

The study was carried out from May 2022 to September 2023 in the Department of Cardiology, Sher-i-Kashmir Institute of Medical Sciences, Srinagar, Jammu and Kashmir, India. Our hospital is a 750-bed tertiary care center and is the primary referral center for ACS in the Union Territory. Acute myocardial infarction (MI) was diagnosed according to the fourth universal definition of MI [7, 8]. In a patient with new ST-segment elevation at the J point in two adjacent leads of ≥0.1 mV in all leads other than V2-V3, an ECG diagnosis of ST-elevation myocardial infarction (STEMI) was made. For leads V2-V3, the criteria were ≥0.2 mV in men aged ≥40 years, ≥0.25 mV in men <40 years, or ≥0.15 mV in women [9]. Informed consent was obtained from each participant before their inclusion. The study was conducted according to the principles outlined in the Declaration of Helsinki and was approved by the institutional review board of the Sher-i-Kashmir Institute of Medical Sciences (approval number: SIMS 131/IEC-SKIMS/2021-352).

Inclusion criteria

The study included all Amarnath pilgrims who were brought to our cardiac facility with a definitive diagnosis of ACS (which was determined through a combination of clinical signs, electrocardiography results, increased levels of biochemical markers for myocardial damage, and echocardiographic findings) [7,8].

Exclusion criteria

Patients were excluded if they refused to give consent to participate in the study.

Data collection

The demographic and clinical data collected included information on age, gender, hypertension, smoking, diabetes mellitus, dyslipidemia, obesity, and family history of coronary artery disease (CAD). Hypertension was determined by the 2018 American Heart Association (AHA)/American College of Cardiology (ACC) guidelines [10]. Diabetes was characterized by a fasting blood glucose level greater than 126 mg/dl, a glycated hemoglobin (HbA1C) level of 6.5 or higher, or if the patient was already being treated for diabetes mellitus. Smoking was considered to be the regular use of tobacco in any form, either currently or within the past year. Dyslipidemia was defined by the presence of any one of the following: low-density lipoprotein (LDL) >130 mg/dl, total cholesterol >200 mg/dl, and high-density lipoprotein (HDL) <40 mg/dl in men and <50 mg/dl in women. Obesity was defined as a body mass index of ≥25 kg/m^2^.

The clinical presentation was noted, which included the type of MI (STEMI or non-STEMI) and the severity of the presentation according to the Killip class. The Killip classification is as follows: Killip class I: no clinical signs of heart failure; Killip class II: the presence of rales (crackles) in the lungs, indicating mild heart failure; Killip class III: pulmonary edema (fluid in the lungs), along with elevated jugular venous pressure and other signs of moderate heart failure; and Killip class IV: cardiogenic shock, with profound hypotension and signs of severe heart failure.

Two-dimensional (2D) echocardiography (Vivid S5 machines with an M3S matrix array probe with a frequency range from 1.7 to 3.2 MHz (GE Vingmed, Horten, Norway)) was performed to assess left ventricular (LV) systolic function and any mechanical complications. The coronary angiographic characteristics, including the site, severity, type, and extent of lesions and the number of vessels involved, were assessed. Stenosis in a vessel (other than the left main coronary artery (LMCA)) was categorized as mild (<50% diameter stenosis), moderate (50%-69% diameter stenosis), and severe (>70% diameter stenosis). The LMCA lesions were classified as non-significant (<50% stenosis) and significant (>50% stenosis). Coronary artery disease was categorized as single vessel disease (SVD), double vessel disease (DVD), or triple vessel disease (TVD) according to the number of major branches with significant involvement. The complexity of atherosclerotic lesions was further categorized according to the joint ACC/AHA task force classification system. The angiographic lesions with 50% stenosis in the LMCA and 70% stenosis in other major vessels (reference vessel diameter of 2.25 mm) were stented. Severe stenosis in smaller vessels (reference vessel diameter <2.25 mm) was left alone.

Procedural details, including vascular access route, site and number of lesions intervened upon, size and number of stents used, periprocedural pharmacotherapy, and use of adjunctive devices, were recorded. Complications and mortality were recorded during the hospital stay, and the factors affecting mortality were investigated. The definition of cardiogenic shock was the presence of a systolic blood pressure of less than 90 mmHg due to myocardial dysfunction caused by ACS in the absence of other causes of shock [11].

The data were compiled and entered into a Microsoft Excel spreadsheet (Microsoft Corp., Redmond, WA), then exported to IBM SPSS Statistics for Windows, version 20.0 (IBM Corp., Armonk, NY) for analysis. Continuous variables were expressed as mean±SD, while categorical variables were summarized as frequencies and percentages. To compare demographic characteristics between groups, we employed independent t-tests for continuous variables such as age and chi-square tests for categorical variables such as gender and clinical presentation types. In assessing the association between clinical parameters and mortality, we utilized Cox regression analysis, adjusting for potential confounding factors such as age, gender, and comorbidities. This allowed us to identify significant predictors of mortality among ACS patients during the pilgrimage. Analysis of variance tests was used for multiple comparisons. A p-value <0.05 was considered statistically significant, and all p-values were two-tailed. To minimize biases and ensure the integrity of the analysis, missing data were systematically handled.

These measures included the establishment of comprehensive data collection protocols and regular data audits. Any missing information was promptly addressed through follow-up with healthcare providers or patients. Double data entry was employed to reduce data entry errors and enhance reliability. Data validation checks were conducted prior to statistical analysis to detect outliers, inconsistencies, and data entry errors. Data cleaning procedures involved identifying and resolving data discrepancies through cross-validation with source documents and patient records. These measures were implemented to minimize the impact of missing data on the study outcomes and ensure the credibility and validity of the findings.

## Results

The total number of patients recruited was 60. The mean age of the study group was 51.19 ± 11.17 years, with 43 (71.7%) males. The mean age at presentation was around a decade later in females compared to males (60.42 ± 11.54 vs. 50.26 ± 12.18 years, p < 0.001).

Risk factors

The factors associated with increased risk were hypertension in 35 (58.3%) patients, smoking in 23 (38.3%) patients, diabetes mellitus in 18 (30%) patients, dyslipidemia in 25 (41.6%) patients, and a family history of CAD in 10 (16.6%) patients. Smoking was more prevalent among males (p =0.001), while diabetes mellitus (p =0.001) was more common in females (Table 1).

**Table 1:** Clinical and demographic profiles of the study population Note: The percentages mentioned are of the total study population. HTN: hypertension; T2DM: type 2 diabetes mellitus; MI: myocardial infarction; CAD: coronary artery disease; AWMI: anterior wall myocardial infarction; IWMI: inferior wall myocardial infarction; HLWMI: high lateral wall myocardial infarction; NSTEMI: non-ST segment elevation myocardial infarction; CAG: coronary angiogram; SVD: single vessel disease; DVD: double vessel disease; TVD: triple vessel disease; LM: left main; LM-TVD: left main with triple vessel disease; non-OB CAD: non-obstructive coronary artery disease The chi-square test for categorical variables (risk factors, types of MI, Killip class, CAG) and the independent sample t-test for continuous variables (age, number, in-hospital mortality) were used. A p-value less than 0.05 was considered statistically significant.

Variable		Total	Males	Females	P-value
Number (percentage)		60	43 (71.7)	17 (28.3)	0.143
Risk factors	Mean age ± SD (range)	51.19 ± 11.17 (37-68) years	50.26 ± 12.18 (37-67) years	60.42 ± 11.54 (48-68) years	0.003
	HTN	35 (58.3%)	23 (38.3%)	12 (20%)	0.035
	T2DM	18 (30%)	6(10%)	12 (20%)	0.001
	Smoking	23 (38.3%)	23 (38.3%)	0	0.001
	Obesity	8 (13.3%)	3 (5%)	5 (8.3%)	0.422
	Family history of CAD	10 (16.6%)	8 (13.3%)	2 (3.3%)	0.232
	Dyslipidemia	25 (41.6%)	13 (21.6%)	12 (20%)	0.864
Type of MI	AWMI	29 (48.3%)	26 (43.3%)	3 (5%)	0.001
	IWMI	15 (25%)	5 (8.3%)	10(16.6%)	0.088
	HLWMI	2 (3.3%)	1 (1.6%)	1 (1.6%)	1.000
	NSTEMI	14 (23.3%)	11 (18.3%)	3 (5%)	0.053
Killip class	Killip I	19 (31.6 %)	13 (21.6%)	6 (10%)	0.116
	Killip II	16(26.6%)	12(20%)	4(6.6%)	0.002
	Killip III	15 (25%)	12(20%)	3(5%)	0.016
	Killip IV	10 (16.6%)	6(10%)	4(6.6%)	0.045
CAG	SVD	17 (28.3 %)	13 (21.6 %)	4 (6.6%)	0.020
	DVD	10 (16.6%)	5 (8.3%)	5 (8.3%)	1.000
	TVD	8 (13.3 %)	6 (10%)	2 (3.3%)	0.302
	LM/ LM-TVD	2 (3.3%)	2 (3.3%)	0	0.309
	Non-OB CAD	9 (15%)	7 (11.6%)	2 (3.3%)	0.283
In-hospital mortality: number (percentage)		9 (15%)	6 (10%)	3 (5%)	0.163

Types of ACS and clinical presentation

The symptoms that were initially reported were chest pain, which was experienced by 50 (83.3%) patients; breathlessness, which was reported by 20 (33.3%) patients; and excessive sweating, which was noted in 12 (20%) patients. Additionally, 40 (66.67%) patients reported that their chest pain radiated into both arms, while six (10%) patients reported that their pain radiated into the right arm, and five (8.3%) patients reported that their pain radiated into the left arm. Out of the 60 patients, 46 (76.66%) had STEMI, while 14 (23.33%) had NSTEMI; STEMI was further divided into anterior wall ST-elevation myocardial infarction (AWSTEMI) in 29 (48.3%), inferior wall myocardial infarction (IWMI) in 15 (25%), and high lateral wall myocardial infarction (HLWMI) in two (3.3%) patients. Among the 60 patients, 19 (31.6%) presented with Killip class I symptoms, 16 (26.6%) had Killip class II symptoms, and 25 (41.6%) presented with advanced Killip class III or IV symptoms (15 (25%) in Killip class III and 10 (16.6%) in Killip class IV). The majority of patients presenting with Killip class III or IV symptoms were males (20 males vs. five females). The average time from the onset of symptoms to hospitalization was 7.6 ± 3.1 hours. This value was significantly higher in patients with Killip class III or IV symptoms (9.3 ± 3.6 hours) compared to those with Killip class I or II symptoms (5.4 ± 2.7 hours, p = 0.01).

Coronary angiographic analysis

We conducted coronary angiography on 46 patients (76.7%). The most common angiographic pattern observed was SVD, which was present in 17 patients (28.3%), followed by DVD in 10 patients (16.6%), and TVD in eight patients (13.3%). Two patients (3.3%) had left main with TVD disease, and nine patients had non-obstructive CAD. Out of the 46 angiographies performed, the most commonly affected vessel was the left anterior descending (LAD), observed in 17 (28.3%) patients, followed by the right coronary artery (RCA) in 13 (21.6%) and the left circumflex (LCX) in five (8.3%) patients. The distribution of CAD patterns across different ACS types showed some variations. Among the patients with STEMI, SVD was observed in 17 (28.3%) cases, while DVD was present in seven (11.6%) cases. Triple-vessel disease occurred in three (5%) STEMI cases. Non-ST-elevation myocardial infarction patients had a different pattern, with DVD in three (5%) cases and TVD in five (8.3%) cases. None had SVD. This indicates that, while SVD was more prevalent in STEMI, TVD was more common in NSTEMI. Based on the ACC/AHA lesion classification system, 28 (46.6%) lesions were classified as type A, 14 (23.3%) as type B, and 4 (6.6%) as type C. In total, 35 (58.3%) patients underwent percutaneous coronary intervention (PCI). Of these, 29 (48.3%) were pharmaco-invasive PCIs, four (6.6%) were primary PCIs, and two (3.3%) were rescue PCIs.

Hospital outcomes

Nine patients (15%) died during their hospital stay, including six males and three females. The number of deaths in the procedural group was four (6.66%), and in the non-procedural group, it was five (8.33%). Of the five deaths in the non-procedural group, four presented with Killip class IV symptoms and one with Killip III symptoms on presentation. The primary causes of death were cardiogenic shock due to severe left ventricular dysfunction in three patients, refractory ventricular tachycardia in one, and ventricular septal rupture in another. In the procedural group, the causes of death were no-reflow in two patients, refractory cardiogenic shock despite revascularization in one and acute stent thrombosis in one patient. In Cox regression analysis (with a total sample size of 60 and nine deaths), advanced Killip class (p = 0.04) and delay in hospitalization of more than six hours (p = 0.03) remained significant predictors of mortality. These findings suggest that patients presenting with advanced Killip class and experiencing delays in hospitalization are at higher risk of adverse outcomes during their hospital stay (Table 2).

**Table 2:** Cox regression analysis of in-hospital mortality HTN: hypertension; T2DM: type 2 diabetes mellitus; AWMI: anterior wall myocardial infarction; CI: confidence interval Cox Regression analysis; a p-value less than 0.05 was considered significant

Variable	Hazard ratio	95% CI	p-value
Male gender	1.38	0.58 - 3.29	0.473
HTN	1.12	0.45 - 2.80	0.805
Type 2 DM	0.88	0.33 - 2.34	0.797
Obesity	0.63	0.17 - 2.35	0.493
AWMI	2.17	0.87 - 5.43	0.098
Advanced Killip class (III, IV)	2.45	0.90 - 6.64	0.04
Delay in hospitalization of ≥6 hours	2.34	0.88- 5.64	0.03

## Discussion

Our research constitutes the initial investigation to elucidate the clinical and demographic characteristics and in-hospital consequences for Amarnath pilgrims diagnosed with ACS. Our findings indicate a notably younger age of onset among these patients, with the majority being male and hypertension serving as the most prevalent risk factor. The majority of these individuals presented with an AWSTEMI, and SVD of the LAD was the most frequent underlying cause. Additionally, we observed that nearly 40% of patients presented with advanced Killip classes (III and IV). The mortality rate was also remarkably high, as 15% of patients died during their hospital stay.

Exposure to high altitudes typically does not cause myocardial ischemia in healthy individuals, but it can pose a significant risk for those with pre-existing cardiovascular conditions or significant cardiovascular risk factors [12]. Although the pathophysiology of ACS at high altitudes is not yet fully understood, experimental studies have shown that coronary reserve is impaired in individuals with significant coronary stenosis due to impaired endothelial vasomotor control and the vasoconstrictive effects of alkalosis caused by increased ventilation and sympathetic activity [13-17]. Our study found that the mean age of presentation for ACS was 51.19 years, which is consistent with established patterns of ACS and reflects a demographic trend that leans toward middle-aged individuals. However, it is noteworthy that the age at presentation for females was approximately a decade later than that of males. The age distribution within the Amarnath pilgrims may be influenced by the pilgrims' unique characteristics and health profiles, contributing to a distinctive age-related presentation. Nevertheless, the younger age of patients contrasts with other pilgrimage studies involving Hajj pilgrimages, which are a decade older [18, 19]. The high prevalence of hypertension, dyslipidemia, smoking, and diabetes among Amarnath pilgrims presenting with ACS emphasizes the need for targeted preventive measures. Hypertension was the most common risk factor, present in almost 60% of study subjects. High altitude activates the sympathetic nervous system, which leads to an increase in cardiac output and sympathetic vasoconstriction, resulting in a modest increase in arterial blood pressure [1]. Our study highlights the importance of tailoring interventions and health education strategies to the prevalent risk factors among pilgrims.

The distribution of patients across different Killip classes was indicative of the severity of clinical presentations. Notably, 40% of our patients were in advanced Killip classes (III/IV), and there was a high mortality rate of 15% among them. The predominance of males (20 males vs. five females) among those presenting with Killip III/IV may be attributed to the fact that men are generally more inclined to undertake rapid ascent. The higher mortality rate among those with advanced Killip classes underscores the significance of timely and appropriate management strategies to mitigate mortality risks. The association between advanced Killip classes and adverse outcomes highlights the importance of prompt intervention. It is noteworthy that patients with Killip III/IV usually experience chest pain while ascending, which can lead to significant delays in getting revascularization due to geographical reasons. The unique circumstances of the Amarnath pilgrimage may further contribute to delays in seeking medical attention, necessitating targeted efforts to enhance awareness, accessibility, and early intervention during the pilgrimage. The average time from onset of symptoms to hospitalization was significantly longer for those with Killip class III/IV (9.3 ±3.6 hours) compared to those with Killip class I/II (5.4 ±2.7 hours), p = 0.01. Thus, we stress the importance of prompt transportation for these patients to improve their outcomes, as delays of more than six hours have been associated with poorer outcomes. The nearest catheterization lab facility is approximately 95 kilometers from the base camp area, and it typically takes two and a half hours to reach it. Our study indicates that individuals who are poorly acclimatized and undertake the holy pilgrimage to the Amarnath cave shrine are at risk of developing severe ACS presentations.

Upon comparing our findings with existing literature on ACS in general and pilgrimage settings [18,19], we have identified unique characteristics among the Amarnath pilgrims. Their distinctive clinical profile, age distribution, and outcomes emphasize the need for pilgrimage-specific research to develop tailored healthcare strategies. Future studies should focus on pilgrimage-specific risk factors and challenges to refine preventive and management approaches. Identifying people at risk of developing ACS and recommending appropriate preventive measures would be highly beneficial in reducing the incidence and severity of ACS among the pilgrims. Unfortunately, there is a general lack of awareness about the cardiovascular risks associated with this journey, which needs to be adequately addressed through intensive educational programs. We recommend optimizing risk factors, such as hypertension, diabetes, and dyslipidemia, before embarking on this holy journey. Our study suggests practical recommendations, including identifying people at risk of developing ACS who are planning this pilgrimage, screening for risk factor profiles, and optimizing risk factors before the journey. We also suggest intensive educational programs to create awareness about the cardiovascular risks associated with this journey. Besides, we also recommend additional medical facilities at the base camp for early identification and timely treatment. We stress the importance of prompt transportation for these patients to improve their outcomes, as a delay of more than six hours was associated with poorer outcomes.

Limitations

While our study was restricted to a single center, it is worth noting that our hospital is a leading cardiac referral center in Union Territory and handles the majority of such cases. Another limitation of our study was the absence of a control group. The study's sample size of 60 patients may be small, but it was chosen based on the unique circumstances of studying ACS among Amarnath pilgrims. To ensure the reliability of the study, several strategies were employed, including pilot studies to refine the study protocol, statistical power analysis to detect clinically meaningful differences, and comprehensive data collection methods. While the study's focus on a specific population may limit its generalizability, it offers valuable insights into ACS management in high-altitude pilgrimage settings. Future research could involve collaborative efforts to overcome sample size limitations. Although observational studies can provide valuable insights, it's crucial to recognize their limitations, including difficulty in determining causality and establishing direct cause-and-effect relationships. Confounding factors like age, comorbidities, and environmental conditions can affect the study results. While the long-term outcomes of these patients were not assessed in our study, it is crucial to establish a more comprehensive database involving other centers in the Union territory to enhance the overall outcomes of these patients.

## Conclusions

The findings of our study offer important information on the clinical and angiographic profiles of ACS in Amarnath pilgrims. The data show that most of our patients were male and presented at a younger age. The age distribution within the Amarnath pilgrim population may be influenced by the pilgrims' unique characteristics and health profiles. Anterior wall STEMI was the most common type of ACS, with 40% of patients presenting in the advanced Killip class (III or IV). Fifteen percent of patients died during their hospital stay. It is clear from the data that there was a significant delay in the presentation of these patients. Possible reasons for hospitalization delays in pilgrims experiencing ACS could be attributed to a multitude of factors. These may encompass issues such as logistical challenges that pilgrims encounter during their travels and a dearth of knowledge regarding the necessity of prompt medical care. It is crucial to recognize these variables in order to develop tailored interventions that aim to minimize delays and enhance the health outcomes for ACS patients who are pilgrims. Advanced Killip class and delays in hospitalization of more than six hours were found to be significant predictors of mortality. It is essential to promptly recognize and manage ACS during pilgrimage. We highlight the importance of implementing interdisciplinary collaboration and community engagement programs to improve healthcare delivery during pilgrimages. It is imperative that measures be taken to improve the outcomes of these patients. The study's findings underscore the necessity for customized healthcare approaches to tackle the particular obstacles faced by Amarnath pilgrims in averting and managing ACS during their spiritual voyage. By focusing on prompt detection, immediate intervention, and maximizing risk factor control, we can strive to lessen the impact of ACS and enhance favorable outcomes for pilgrims embarking on this sacred pilgrimage. 
